# The influences of smartphone use on the status of the tear film and ocular surface

**DOI:** 10.1371/journal.pone.0206541

**Published:** 2018-10-31

**Authors:** Jung Han Choi, Ying Li, Seon Ho Kim, Rujun Jin, Yung Hui Kim, Won Choi, In Cheon You, Kyung Chul Yoon

**Affiliations:** 1 Department of Ophthalmology, Chonnam National University Medical School and Hospital, Gwangju, Korea; 2 MSC Research Institute and Kim Seon Ho Eye Clinic, Gwangju, Korea; 3 Department of Biomedical Sciences and Centers for Creative Biomedical Scientists at Chonnam National University, Gwangju, Korea; 4 Department of Ophthalmology, Chonbuk National University Medical School and Hospital, Jeonju, Korea; Boston University School of Medicine, UNITED STATES

## Abstract

**Purpose:**

To investigate the influences of smartphone use on ocular symptoms, status of the tear film, and oxidative stress indices in the tears and at the ocular surface.

**Methods:**

Eighty healthy volunteers were enrolled in the study. Subjective symptoms and asthenopia were evaluated using the ocular surface disease index (OSDI), visual analogue scale (VAS), and computer vision syndrome (CVS) score before and after smartphone or computer display (control) use. The status of the tear film was evaluated using fluorescein film break-up time (FBUT), non-invasive keratograph break up time (NIKBUT), Schirmer score, keratoepitheliopathy (KEP), and tear meniscus height (TMH). Oxidative stress markers in the tear film including hexanoyl lysine (HEL), 4-hydroxy-2-nonenal (4-HNE), malondialdehyde (MDA), and 8-oxo-2’-deoxyguanosine (8-OHdG) in the tear film were measured using ELISA. Reactive oxygen species (ROS) at the ocular surface were measured through 2’,7’-dichloro-dihydrofluorescein diacetate. All measurements were conducted at baseline, and after use for 1 and 4 h.

**Results:**

All parameters showed no significant group-wise differences at baseline. Scores of OSDI, VAS, fatigue, burning sensation, and dryness showed significant increases after 1 and 4 h of smartphone use compared with those at baseline (all *P* < 0.05). The smartphone group showed higher OSDI, fatigue, burning, and dryness scores than the control group at 4 h. Smartphone use showed significantly decreased FBUT and NIBUT at 4 h than those at baseline (*P* < 0.01). In the smartphone group, the concentration of HEL significantly increased at 4 h compared with that at baseline and 1 h (*P* < 0.01). Both groups showed increased ROS with higher value in the smartphone group versus the control group at 4 h (*P* < 0.01).

**Conclusions:**

Smartphone use could not only aggravate subjective symptom indices such as the OSDI, VAS, and CVS but also induce tear film instability and oxidative stress indices in the tears and at the ocular surface.

## Introduction

Visual display terminal (VDT) use is increasingly common not only in VDT workers but also in the general population due to the widespread use of mobile devices and smart phones.[1] Smartphone use has significant impact on daily life activity. Smartphones enable varied activities including browsing the web, watching video, group chatting, and social networking as compared to those in the previous generation. Therefore, time spent viewing at display screens has increased with the use of smartphone use than ordinary cellular phone. One study reported that the average time spent using a smartphone nearly doubled from 98 minutes per day in 2011 to 195 minutes in 2013.[2]

Previous studies reported that ordinary cellular phones affect human health as well as daily life. Cellular phone use correlates with many health problems such as sleep disorder, headaches, leukemia, brain tumors and malignant melanoma of the eyes.[3,4] With the increasing use of smartphones, recent studies have reported an association between ocular health and smartphone use. One study reported two cases of transient monocular vision loss associated with smartphone use.[5] Excessive use of smartphone also led to acute acquired comitant esotropia in adolescents.[6] A study including subjects with pediatric dry eye disease (DED) reported that the rate and mean time spent using smartphones were greater in the DED than the non-DED group.[7] Because increased time of use of smartphone is related to DED, excessive use of smartphones may affect the tear film and the ocular surface. Office workers who spent more than 4 h watching VDT experienced severe ocular symptoms, similarly, excessive smartphone use has been associated with multiple ocular symptoms.[3,8] Our recent study indicated that blue light emitted from the smartphone screen had adverse effect on the corneal epithelial cells in humans.[9] Overexposure to blue light caused deterioration of the tear film and increased levels of inflammatory markers and reactive oxygen species (ROS) production at the ocular surface of mice.[10]

To the best of our knowledge, any study related to the ocular symptoms, signs or oxidative stress indices at the tears or the ocular surface associated with smartphone use has not been reported yet. In the present study, we investigated the comparative effects of the use of smartphone and computer display on the subjective ocular asthenopia, tear film status, and oxidative marker levels in healthy subjects.

## Materials and methods

### Study population

**Fig 1:**
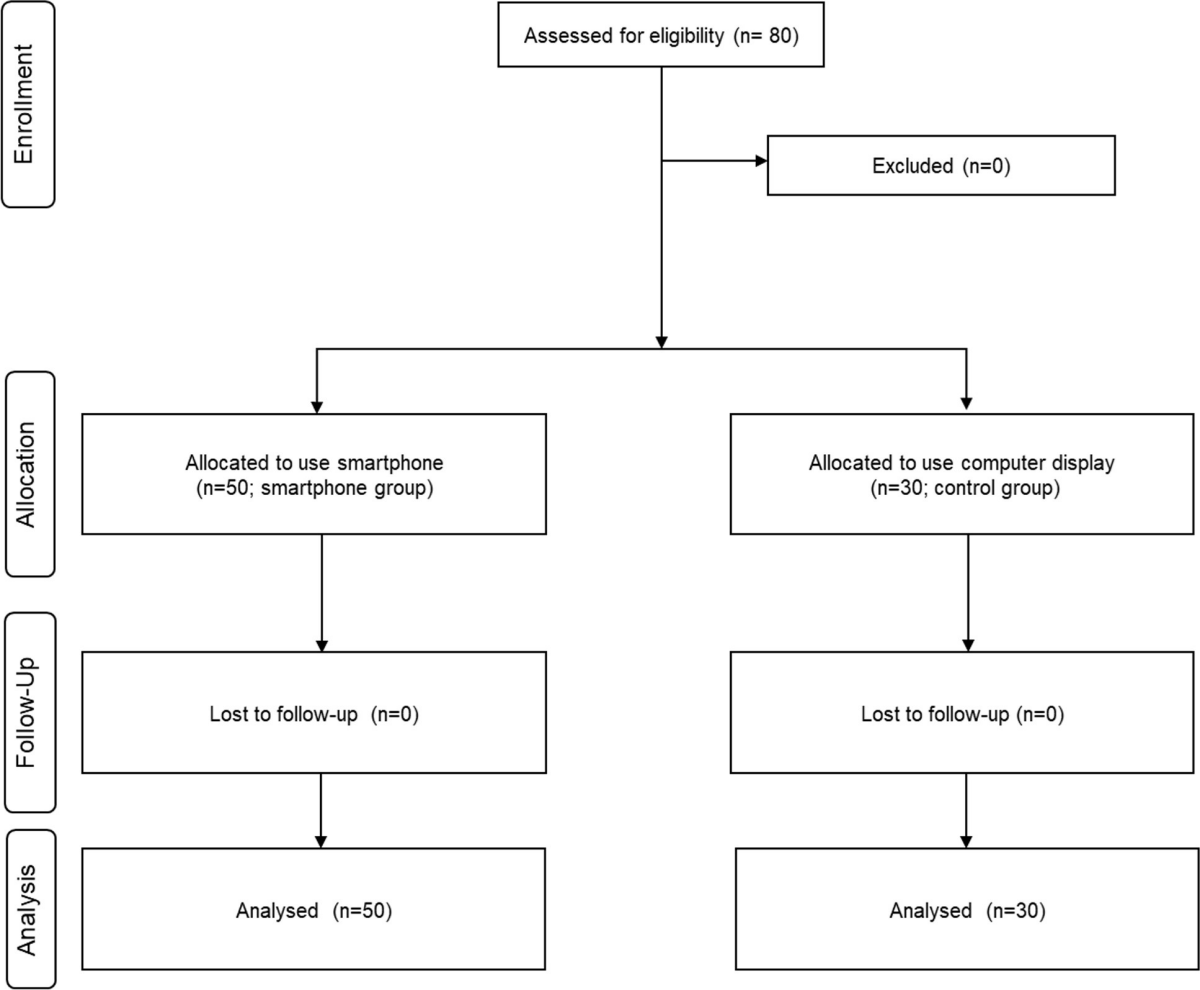
Flow chart showing subject enrollment, allocation, follow up, and analysis.

This study was a prospective, nonrandomized, comparative clinical study to evaluate the effects of smartphone and computer display usage on subjective symptoms and changes in the tear film and ocular. Eighty volunteers who were healthy adults without ocular disease, systemic disease which could affect ocular condition, contact lens use, or surgical history were included. Subjects who used eye drops or were pregnant at the time of the study were excluded. The study was conducted in accordance with the Declaration of Helsinki. Written informed consent was obtained from all subjects, and the protocol was approved by the Institutional Review Board of Chonnam National University Hospital. The study was registered with The ISRCTN registry. (ISRCTN17257070) The report of the study followed the TREND guideline (S1 TREND Checklist). Study protocols are included as Supplemental files (S1 and S2 Protocols.) The healthy volunteers were assigned to either the smartphone (n = 50) or computer display (n = 30; control) groups. A flow diagram of the participants is shown in Fig 1. In the smartphone group, subjects used the same smartphone with a 5.1 in light emitting diode (LED) screen from the same manufacturer (Galaxy S6, Samsung, Seoul, South Korea). In the control group, subjects used the same computer display with a 19.0 in screen (Samsung). The illumination intensity was fixed at 80% of maximum brightness. The distance and angle between the screen of display and the subjects was limited to constant value. All subjects were asked to play a puzzle game.

### Assessment of subjective ocular symptoms and asthenopia

All examinations were performed in 20 subjects per day, for 4 d, before and after 1 h and 4 h of smartphone or computer display use, by a single investigator (KCY). Examination was conducted in the same outpatient clinic with constant temperature and humidity at the same time of day. Parameters of the tear film and ocular surface were evaluated within 15 min in the following order: Non-invasive keratograph break-up (NIKBUT), tear meniscus height (TMH), fluorescein break-up time (FBUT), keratoepitheliopathy (KEP) score, Schirmer test value, and tear sampling data. In all subjects, testing time and sequence was per standardized protocol. After examination at baseline and 1 h, subjects started viewing the smartphone or computer display.

Subjective ocular symptoms and asthenopia were evaluated using the ocular surface disease index (OSDI), visual analogue scale (VAS), and computer vision syndrome (CVS) score before and after use for 1 h and 4 h of use. The OSDI questionnaire included the following subscales: (1) Ocular symptoms (OSDI symptoms), (2) vision-related activities in daily living (OSDI visual function), and (3) environmental triggers (OSDI triggers).[11] The total OSDI score and each subscale score, ranging from 0 to 100, were analyzed.[11] Changes in ocular fatigue before and after smartphone use were examined by means of VAS test to assess subjective asthenopia. We used a form with a scale of 0, I have no fatigue to 100, I feel extremely fatigued marked on the extreme ends of a 100-mm line.[12] We used the modified questionnaire of Ames et al.[13] to evaluate the CVS score. The questionnaires included five questions on the severity of fatigue, burning, dryness, blurred vision, and dullness associated with subjective asthenopia; each question was graded on a numerical scale of 0–6, with 0 defined as none and 6 as most severe. All subjects completed the questionnaire before use and immediately after 1 h and 4 h use.

### Assessment of status of the tear film

FBUT and Schirmer score were measured as previously described.[14] FBUT was evaluated 2 min after instillation of 2 mL of 0.5% fluorescein (Alcon, Fort Worth, TX, USA). Subjects were subsequently asked to blink several times. The time in seconds between the last complete blink and the appearance of the first corneal black spot was measured three times, and the mean value was recorded. The Schirmer test was performed 5 min after instillation of a 10 μL of 0.5% fluorescein (Alcon) and 0.5% proparacaine hydrochloride, dropwise in the conjunctival sac. A standard Schirmer test strip was then placed in the lateral canthus for another 5 min with the eyes closed. The length of strip wetting was measured using the millimeter scale. Keratoepitheliopathy was scored by multiplying the area score by density score after staining with 0.5% fluorescein dye. The staining area was graded on a numerical scale of 0–3, with 0 representing no punctate staining; 1, less than one-third; 2, one-third to two-thirds; and 3, more than two-thirds. The staining density was graded on a numerical scale of 0–3, with 0 representing no punctate staining; 1, sparse density; 2, moderate density; and 3, high density with overlapping lesions.[15]

Non-invasive keratograph break-up (NIKBUT) and tear meniscus height (TMH) were measured using Keratograph 5M (Oculus GmbH, Wetzlar, Germany). All measurements were performed three times, before and immediately after 1 h and 4 h smartphone or computer display use.[16–18] Room temperature and humidity were maintained at 20–25°C and 30–40%, respectively.[17,18] NIKBUT was evaluated using an infrared diode video program in Keratograph 5M. The NIKBUT-average (average time of all tear film breakups) was analyzed. TMH was evaluated using four infrared diodes under deactivation of red ring illumination, and graded perpendicular to the lid margin at the 6 o’clock position of the corneal midline.[19]

### Tear collection

Basal tear samples were carefully obtained to avoid touching the ocular surfaces from the inferior tear meniscus of both eyes by using glass capillary tubes (Corning, Inc., Corning, NY, USA) or micropipettes (Eppendorf, Hamburg, Germany) before and immediately after 1 h and 4 h of smartphone use as described previously.[20] Twenty microliter tear samples were obtained and diluted with phosphate-buffered saline. Tear samples were placed in microtubes and stored at −70°C until further examination.

### Measurement of oxidation markers using ELISA

Total protein levels of the oxidation stress markers, hexanoyl lysine (HEL, JaICA, Haruoka, Japan), 4-hydroxy-2-nonenal (4-HNE, Cell Biolabs, San Diego, CA, USA), malondialdehyde (MDA, Cell Biolabs), and 8-oxo-2’-deoxyguanosine (8-OHdG, Cell Biolabs), in the subject`s tears were detected using ELISA according to the manufacturer’s instructions. Tear concentration of HEL was measured using commercially available HEL ELISA, as reported previously.[21] Protein samples (100 μL, 10 μg/mL) of 4-HNE, MDA, and 8-OHdG were absorbed onto a 96-well plate for 2 h at 37°C. The minimal detectable concentrations of HEL, 4-HNE, MDA and 8-OHdG were above 2.6 nmol/L, 0.078 μg/mL, 2 pmol/mg, and 100pg/mL, respectively.

### Measurement of cellular reactive oxygen species production through conjunctival Impression cytology

The 2’,7’-dichlorodihydrofluorescein diacetate (DCF-DA) assay kit, a cellular reactive oxygen detection kit, was used to measure cellular ROS production according to the manufacturer’s protocol. DCF-DA is a non-fluorescent and membrane permeable compound that becomes fluorescent and membrane impermeable after oxidation.[10] Cell collection to evaluate ROS level through impression cytology was performed as follows: A piece of cellulose acetate filter paper (MFS membrane filters, Advantec MFS, Dublin, CA), approximately 6.2-mm in diameter, was applied, dull side down, to the lower nasal bulbar conjunctiva adjacent to the corneal limbus under topical anesthesia with 0.5% proparacaine hydrochloride. An investigator pressed the filter gently with blunt, smooth-tipped forceps for 5 to 10 s. The paper was immediately placed into a well of a 96-well plate containing 200 μL of Krebs-Ringer bicarbonate buffer. The cells were incubated in the dark with 20 μg/mL of 2’7’-dichlorofluorescein for 30 min at 37°C. The plates were read at excitation of 480 nm and emission of 530 nm (FACSCalibur cytometer; BD Biosciences, San Jose, CA, USA).[22]

### Statistical analysis

Statistical Package for the Social Sciences software version 18.0 (SPSS, Inc, Chicago, IL, USA) was used for all statistical analyses. Data are presented as the mean ± standard deviation. Wilcoxon signed rank test was used to assess changes in the various parameters before and after 1 h and 4 h of smartphone or computer display use. Mann-Whitney U test was used to compare results between the two groups. Differences were considered statistically significant at p value of less than 0.05.

## Results

### Participants

The mean age of the 80 healthy subjects was 25.96 ± 2.98 y (range, 21–36 y), and 30 were female individuals. Subject demographics and tear film parameters are presented in Table 1.

**Table 1:** Baseline characteristics of participants.

Characteristics	Smartphone group (N = 50)	Control group (N = 30)
Age (y)	25.52 ± 2.92	26.70 ± 2.98
Sex (male/female)	33/17	17/13
TBUT (s)	6.76 ± 2.03	6.35 ± 2.30
NIKBUT (s)	10.26 ± 6.13	11.84 ± 6.97
Schirmer test (mm)	13.66 ± 4.10	13.85 ± 3.15
KEP (0–9)	0.26 ± 0.54	0.35 ± 0.59
TMH (mm)	0.20 ± 0.05	0.21 ± 0.05

Data are expressed as the mean ± standard deviation.

TBUT, tear break up time; NIBUT, non-invasive keratograph break up time; KEP, keratoepitheliopathy; TMH, tear meniscus height.

### Subjective ocular symptoms and asthenopia

**Fig 2:**
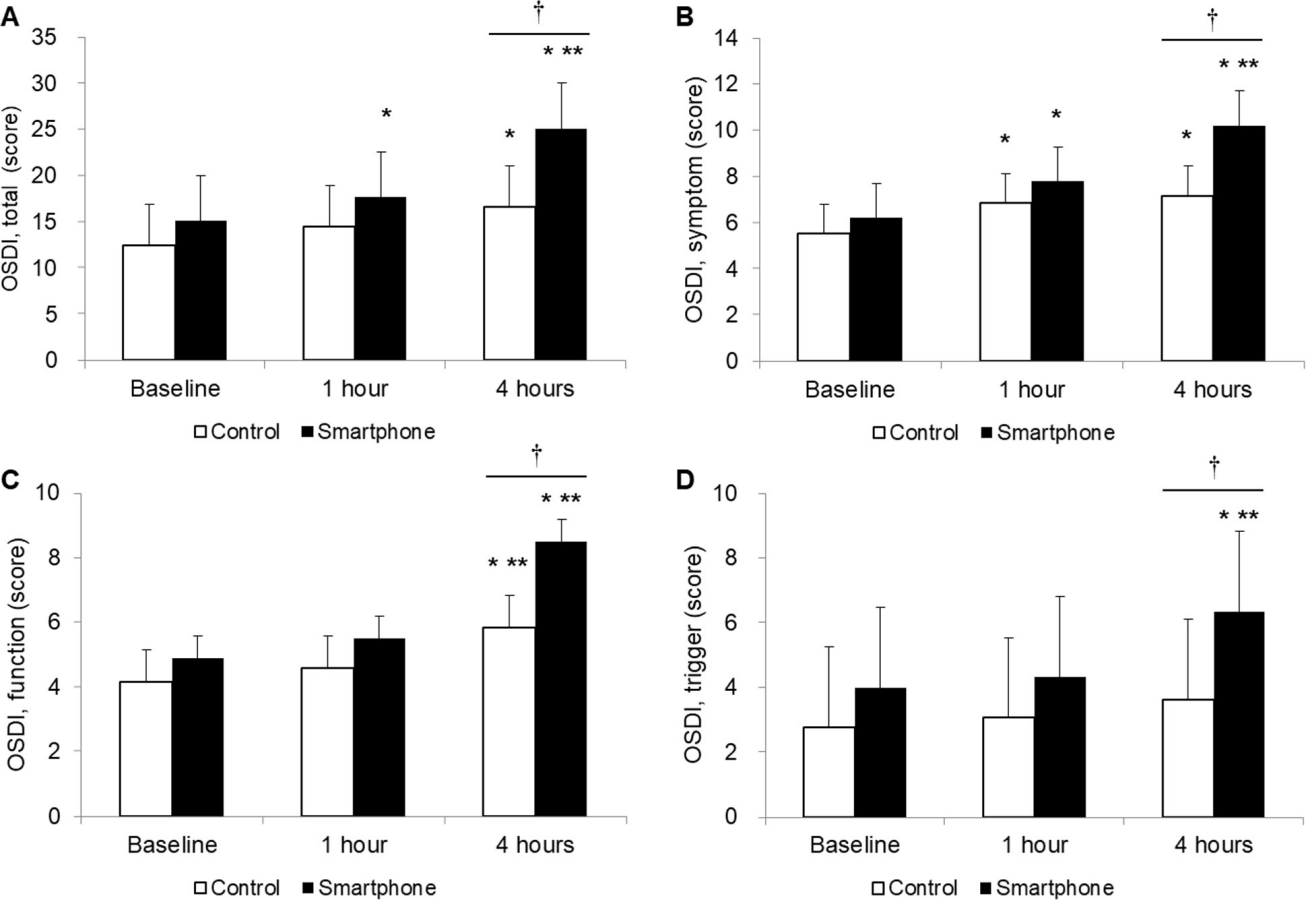
Changes in the ocular surface disease index (OSDI) scores. OSDI total (A), OSDI symptom (B), OSDI visual function (C), and OSDI trigger (D). *P < 0.05 versus baseline. **P < 0.05 versus 1 h. ^†^*P* < 0.05 between the two groups.

The total OSDI score at baseline was 15.08 ± 8.83 in the smartphone group and 12.44 ± 7.55 in the control groups. The total OSDI score increased to 17.63 ± 7.74 at 1 h (*P* < 0.01 vs. baseline) and 25.03 ± 10.61 at 4 h (*P* < 0.01 vs. baseline and 1 h) in the smartphone group, and 14.47 ± 7.29 at 1 h (*P* = 0.68 vs. baseline) and 16.61 ± 6.45 at 4 h (*P* < 0.01 vs. baseline and *P = 0*.*52* vs. 1 h) in the control group. In the smartphone group, the OSDI symptom, visual function and trigger scores increased to 7.80 ± 3.22 (*P* < 0.01 vs. baseline), 5.50 ± 3.49 (*P* = 0.10 vs. baseline) and 4.33 ± 4.21 (*P =* 0.32 vs. baseline) at 1 h, and 10.20 ± 5.25 (*P* < 0.01 vs. baseline and 1 h), 8.50 ± 5.47 (*P* < 0.01 vs. baseline and 1 h), and 6.33 ± 5.47 (*P* < 0.01 vs. baseline and 1 h) at 4 h, respectively. At 4 h, the OSDI total, symptom, visual function, and trigger scores were higher in the smartphone group than in the control group (Fig 2).

**Fig 3:**
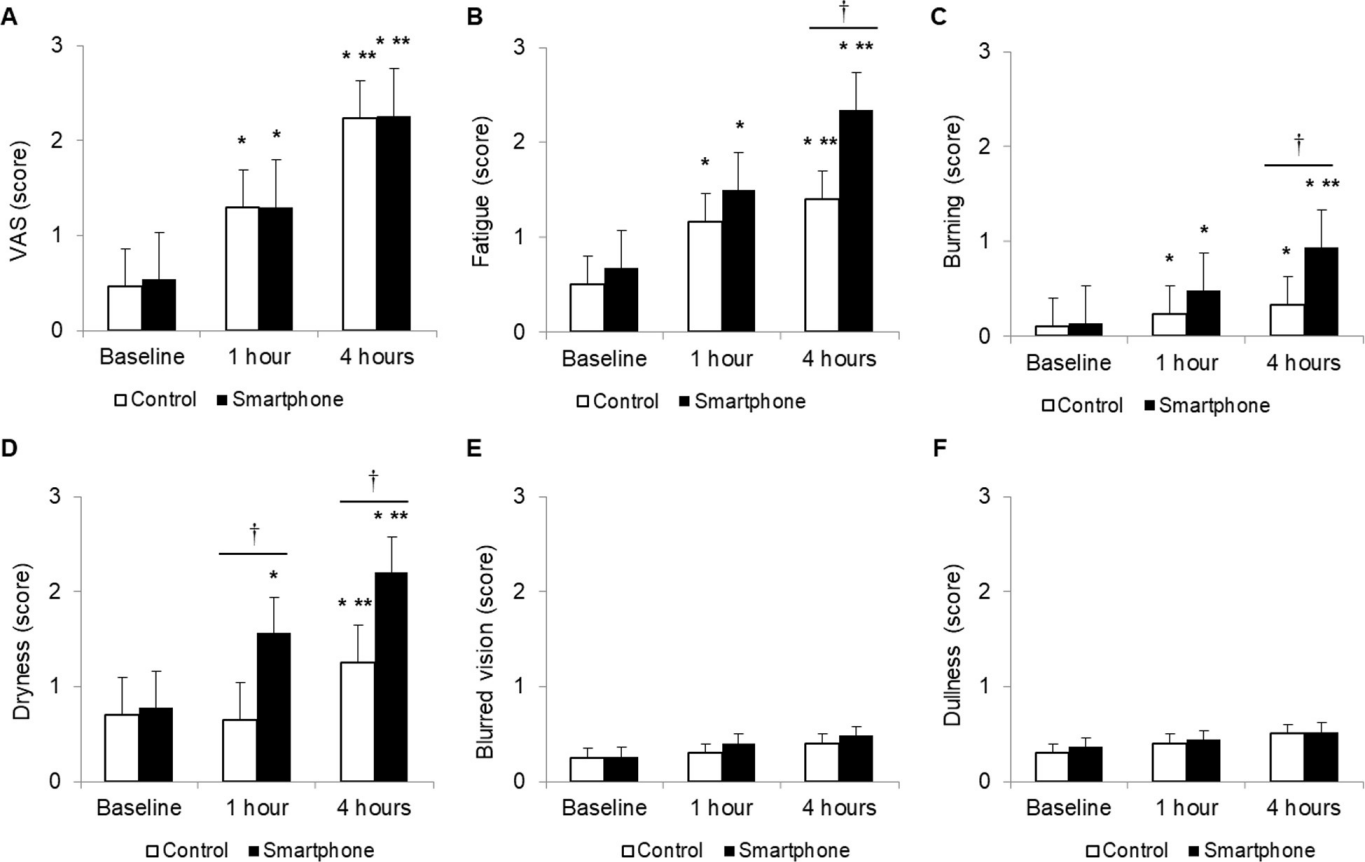
**Changes in visual analogue scale (A) and computer vision syndrome scores including fatigue (B), burning (C), dryness (D), blurred vision (E), and dullness (F).** *P < 0.05 versus baseline. **P < 0.05 versus 1 h. ^†^*P* < 0.05 between the two groups.

VAS in the smartphone and control groups was 0.54 ± 0.68 and 0.46 ± 0.75 at baseline, 1.30 ± 0.74 (*P* < *0*.*01* vs. baseline) and 1.30 ± 0.88 (*P* < *0*.*01* vs. baseline) at 1 h, and 2.26 ± 0.75 (*P* < *0*.*01* vs. baseline and 1 h) and 2.33 ± 0.90 (*P* < *0*.*01* vs. baseline and 1 h) at 4 h. There was no significant difference in VAS between the two groups (Fig 3A).

CVS scores at baseline for fatigue, burning, dryness, blurred vision, and dullness in the smartphone and control groups were 0.68 ± 0.71 and 0.50 ± 0.51; 0.14 ± 0.40 and 0.10 ± 0.31; 0.78 ± 0.99 and 0.73 ± 0.45; 0.26 ± 0.69 and 0.23 ± 0.43; and 0.36 ± 0.77 and 0.33 ± 0.48, respectively. In the smartphone group, the fatigue, burning, and dryness scores at 1 h were 1.50 ± 0.92 (*P* < 0.01 vs. baseline), 0.48 ± 0.73 (*P* < 0.01 vs. baseline) and 1.56 ± 1.20 (*P* <0.01 vs. baseline), and the respective scores at 4 h were 2.34 ± 1.29 (*P* < 0.01 vs. baseline and 1 h), 0.94 ±1.13 (*P* < 0.01 vs. baseline and 1 h) and 2.20 ± 1.53 (*P* < 0.01 vs. baseline and 1 h). The fatigue, burning, and dryness scores were higher in the smartphone groups than in the control group at 4 h (all *P* < 0.05). However, blurred vision and dullness scores showed no significant changes related to smartphone or computer display use, with no significant difference between the two groups (Fig 3B–3F).

### Status of the tear film

**Fig 4:**
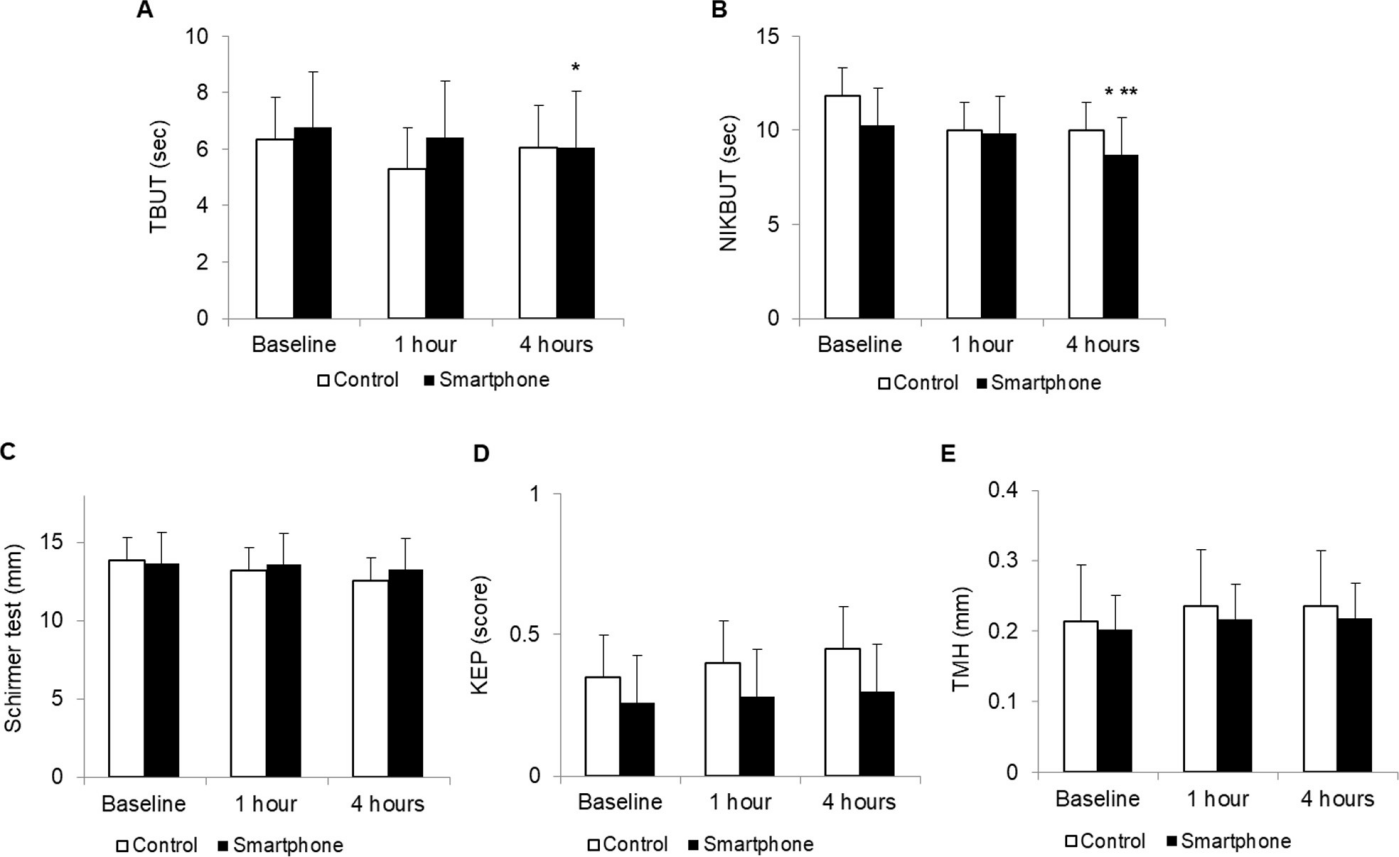
**Changes in tear break up time (TBUT, A), non-invasive kertograph break up time (NIKBUT, B), Schirmer test (C), keratoepithelioapthy (KEP, D) and tear meniscus height (TMH, E).** *P < 0.05 versus baseline. **P < 0.05 versus 1 h.

At baseline, FBUT and NIKBUT were 6.76 ± 2.03 and 10.26 ± 6.13 s in the smartphone group, and 6.35 ± 2.30 and 11.84 ± 6.97 s in the control group. In the smartphone group, FBUT and NIKBUT decreased to 6.42 ± 1.74 (*P* = 0.11 vs. baseline) and 9.85 ± 5.05 s (*P* = 0.11 vs. baseline) at 1 h, and 6.06 ± 1.92 (*P* < 0.01 vs. baseline, *P* = 0.08 vs. 1 h) and 8.72 ± 4.79 s (*P* < 0.01 vs. baseline, *P* = 0.02 vs. 1 h) at 4 h. However, no significant change was noted in the Schirmer test value, keratoepitheliopathy scores, or TMH after smartphone or computer display use. There were no significant differences in tear film parameters between the two groups (Fig 4).

### Oxidation markers in the tear film

**Fig 5:**
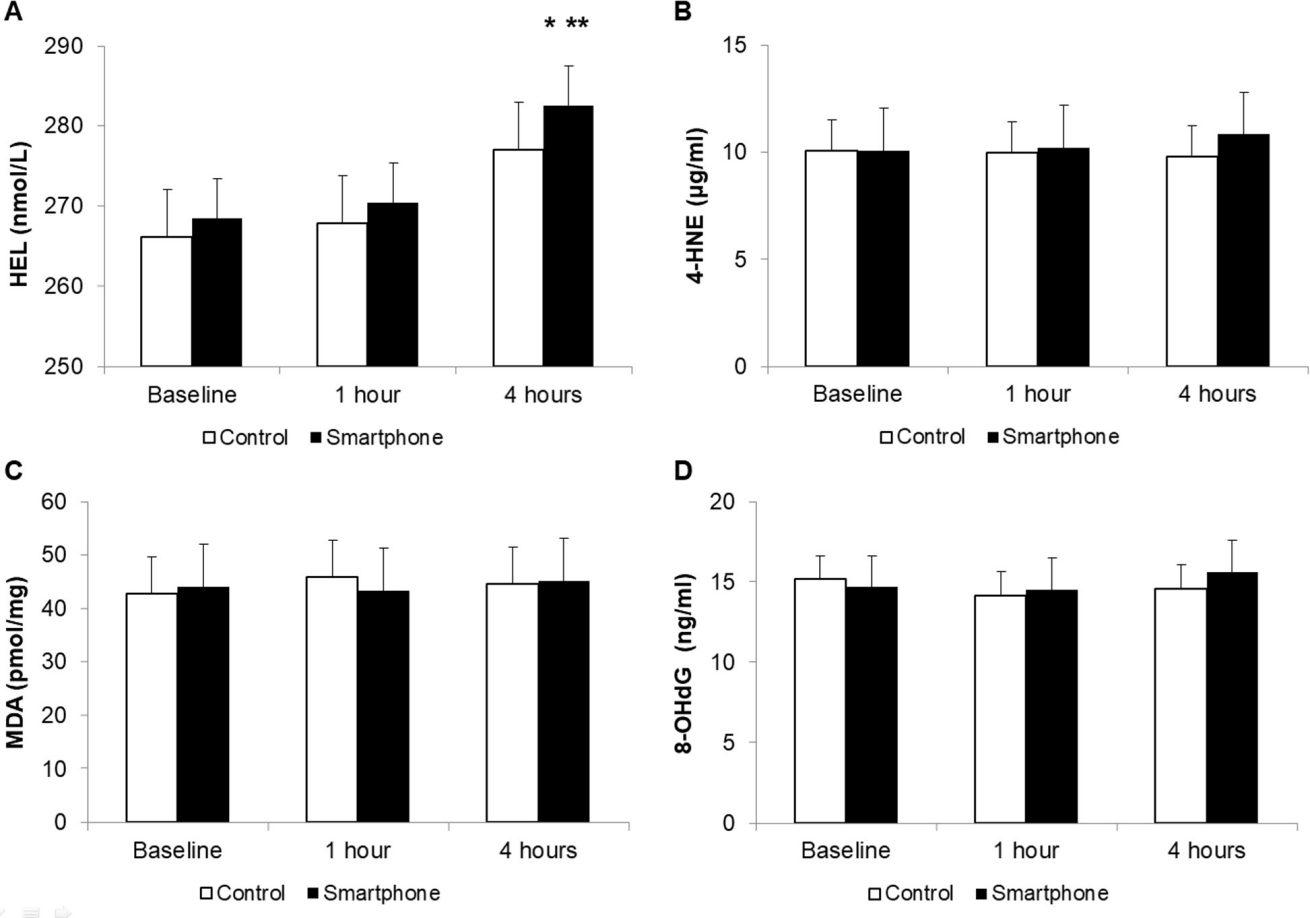
**Concentrations of hexanoyl lysine (HEL, A), 4-hydroxy-2-nonenal (4-HNE, B), malondialdehyde (MDA, C) and 8-oxo-2’-deoxyguanosine (8-OHdG, D) in the tear film.** * *P* < 0.05 versus baseline. ** *P* < 0.05 versus 1 h.

The basal concentrations of HEL, 4-HNE, MDA, and 8-OHdG in the smartphone and the control groups were 268.49 ± 19.98 and 266.08 ± 26.96 nmol/L, 10.08 ± 3.07 and 9.76 ± 4.68 μg/mL, 44.01 ± 6.03 and 41.90 ± 11.22 pmol/mg, and 14.69 ± 4.17 and 15.28 ±1.30 ng/ml, respectively. The HEL concentration post smartphone use was 270.40 ± 17.04 nmol/L (*P* = 0.78 vs. baseline) at 1 h and 282.53 ± 14.08 nmol/L (*P* < 0.01, vs. baseline and 1 h) at 4 h. There was no significant difference in the HEL concentration between two groups. The concentrations of 4-HNE, MDA, and 8-OHdG showed no significant change after smartphone or computer display use. (Fig 5).

### Cellular reactive oxygen species production at the ocular surface

**Fig 6:**
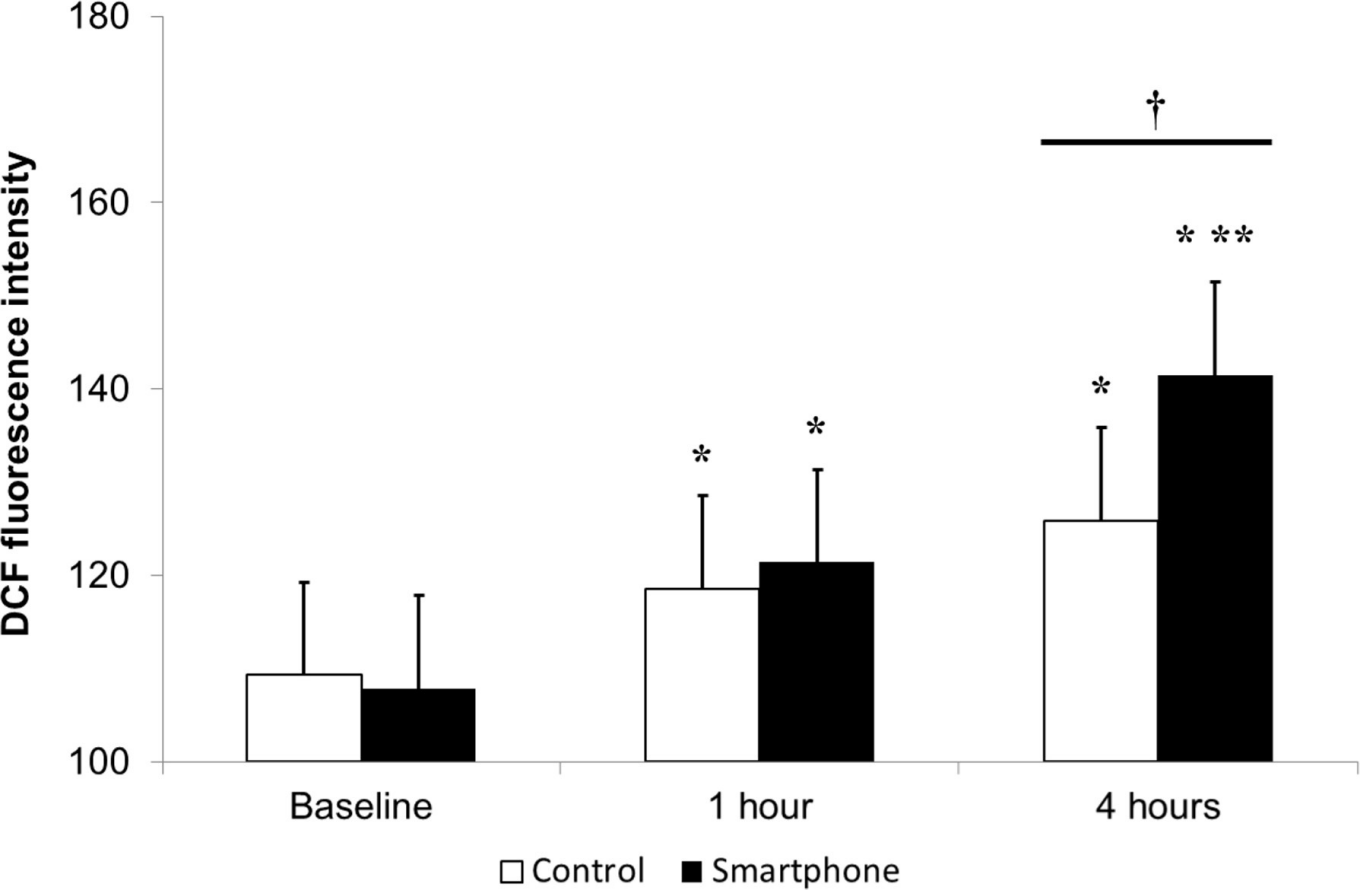
Reactive oxygen species production in the conjunctival epithelium afater smartphone use measured through 2’,7’-dichlorodihydrofluorescein diacetate. *P < 0.05 versus the baseline value. ^†^*P* < 0.05 between the two groups.

To assess net oxidative stress, ROS levels in the conjunctiva epithelium were measured using a DCF-DA assay kit. At baseline, there was no significant difference in DCF-DA fluorescein intensity between the two groups (107.90 ± 27.54 and 108.73 ± 14.48 in the smartphone and control groups). DCF-DA fluorescein intensity showed significant increased at 1 and 4 h in both groups, with higher value in the smartphone group versus the control group at 4 h (141.56 ± 22.39 vs. 123.03 ± 18.45 in the smartphone and control groups, *P* < 0.01) (Fig 6). Table 2 shows the parameters of both groups during VDT use.

**Table 2:** Changes in the ocular surface disease index scores, visual analogue scale, computer vision syndrome scores, status of the tear film, oxidative stress markers, and dichlorodihydrofluorescein fluorescein intensity production after use of the smartphone and computer display (control).

Parameters	Smartphone group (N = 50)			Control group (N = 30)		
	Baseline	1 h	4 h	Baseline	1 h	4 h
**OSDI (score)**						
**Total**	15.08 ± 8.83	17.63 ± 7.74*	25.03 ± 10.61* ** ^†^	12.44 ± 7.55	14.47 ± 7.29	16.61 ± 6.45*
**Symptom**	6.20 ± 3.58	7.80 ± 3.22*	10.20 ± 5.25* ** ^†^	5.50 ± 3.31	6.83 ± 2.45*	7.17 ± 2.84*
**Visual function**	4.88 ± 3.41	5.50 ± 3.49	8.50 ± 5.47* ** ^†^	4.17 ± 3.42	4.58 ± 2.81	5.83 ± 2.81* **
**Trigger**	4.00 ± 4.21				4.33 ± 4.21	6.33 ± 5.47* ** ^†^	2.78 ± 4.00	3.06 ± 4.08	3.61 ± 4.74
**VAS (score)**	0.54 ± 0.68	1.30 ± 0.74*	2.26 ± 0.75* **	0.46 ± 0.75	1.30 ± 0.88*	2.23 ± 0.90* **
**CVS (score)**						
**Fatigue**	0.68 ± 0.71	1.50 ± 0.92*	2.34 ± 1.29* ** ^†^	0.50 ± 0.51	1.17 ± 1.18*	1.40 ± 1.00* **
**Burning**	0.14 ± 0.40	0.48 ± 0.73*	0.94 ± 1.13* ** ^†^	0.10 ± 0.31	0.23 ± 0.43*	0.33 ± 0.48*
**Dryness**	0.78 ± 0.99	1.56 ± 1.20* ^†^	2.20 ± 1.53* ** ^†^	0.73 ± 0.45	0.70 ± 0.65	1.30 ± 0.91* **
**Blurred vision**	0.26 ± 0.69	0.40 ± 0.53	0.38 ± 0.53	0.23 ± 0.43	0.30 ± 0.47	0.33 ± 0.48
**Dullness**	0.36 ±0.77	0.44 ± 0.61	0.52 ± 0.71	0.33 ± 0.48	0.43 ± 0.50	0.43 ± 0.50
**Tear film status**									
**TBUT (s)**	6.76 ± 2.05	6.42 ± 1.74	6.06 ± 1.92*	6.35 ± 2.30	6.94 ± 1.67	6.05 ± 1.73
**NIKBUT (s)**	10.26 ± 6.13	9.85 ± 5.05	8.72 ± 4.79* **	11.84 ± 6.97	9.98 ± 5.54	9.99 ± 5.46
**Shirmer test** **(mm)**	13.64 ± 2.85				13.58 ± 3.80	13.26 ± 3.21	13.85 ± 3.15	13.15 ± 3.36	12.50 ± 2.59
**KEP (0–9)**	0.26 ±0.56				0.28 ± 0.54	0.3 ± 0.58	0.35 ± 0.59	0.40 ±0.60	0.45 ± 0.60
**TMH (mm)**	0.20 ± 0.05				0.22 ± 0.56	0.22 ± 0.08	0.21 ±0.05	0.24 ± 0.50	0.24 ± 0.12
**Oxidative stress markers**									
**HEL** **(nmol/L)**	268.49 ± 19.98	270.40 ± 17.04	282.53 ± 14.08* **	266.08 ± 26.96	267.83 ± 39.97	277.02 ± 54.04
**4-HNE (μg/mL)**	10.08 ± 3.07	10.22 ± 3.03	10.54 ± 3.32	9.76 ± 4.68	9.64 ± 3.36	9.68 ± 2.30
**MDA (pmol/mg)**	44.01 ± 6.03	43.38 ± 4.71	45.14 ± 9.34	41.90 ± 11.22	45.48 ± 14.62	44.73 ± 9.45
**8-OHdG (ng/ml)**	14.69 ± 4.16	14.51 ± 4.64	15.61 ± 5.35	15.28 ± 1.30	14.45 ± 1.99	14.95 ± 1.65
**DCF fluorescein intensity**	107.90 ± 27.54	121.42 ± 28.31*	141.56 ± 22.39* ** ^†^	108.73 ± 14.48	118.37 ± 10.49*	123.03 ± 18.45*

Data are expressed as mean ± standard deviation.

OSDI, ocular surface disease index; VAS, visual analogue scale; CVS, computer vision syndrome; TBUT, tear break-up time; NIKBUT, non-invasive keratograph break-up time; KEP, keratoepitheliopathy; TMH, tear meniscus height; HEL, hexanoyl lysine; 4-HNE, 4-hydroxy-2-nonenal; MDA, malondialdehyde; 8-OHdG, 8-oxo-2’-deoxyguanosine; DCF, dichlorodihydro-fluorescein.

*P < 0.05 versus baseline.

**P < 0.05 versus 1 h.

†P < 0.05 between the two groups

## Discussion

Recent studies have reported various adverse effects of smartphone use on ocular health.[3,5–7] As the use of smartphone becomes more widespread and integrated in daily activity, ocular health problems due to smartphone use becomes an increasingly serious issue. Our prospective nonrandomized comparative clinical study investigated the influence of excessive smartphone use on the tear film and ocular surface as compared with that of computer display use.

To evaluate subjective symptom changes, we analyzed the OSDI, VAS, and CVS scores. In the present study, OSDI scores indicating dry eye symptom severity significantly increased, whereas FBUT and NIKBUT decreased after smartphone use. In addition, the smartphone group showed higher total OSDI, symptom, visual function, and trigger scores at 4 h than the computer display group. Dry eye-like symptoms, such as irritation, burning, and dryness, are common in people working at VDT screens.[23] Uchino and colleagues observed that VDT workers had short tear break up time and increased corneal fluorescent staining, despite normal lacrimal function.[1] Excessive evaporation of the tear fluid due to prolonged blinking intervals while gazing is considered as a causative factor in VDT-associated dry eye.[24, 25] The high cognitive demands associated with reading tasks, such as using a VDT or reading text messages, led to reduction in the spontaneous eye-blink rate.[26] Furthermore, the incomplete blink frequency and the exposure of the ocular surface area increase under VDT use.[23] As a result of smartphone use, harmful factors including the decreased blink rate, frequent incomplete eye closure and increased ocular surface exposure may disturb the delicate homeostatic balance of the ocular surface system, inducing subjective symptoms and tear instability.[27] It is likely that the decline of FBUT and NIKBUT is associated with these adverse factors from smartphone use.

Symptoms such as eye fatigue, aching in and around the eyes, blurred vision and headache are collectively referred to as asthenopia.[28] Asthenopia is the ocular component of CVS.[29] Almost 60 million people around the world suffer from CVS, and one million new cases are estimated to occur annually.[30] CVS includes ocular, visual and musculoskeletal symptoms that result from excessive VDT use.[29] The severity of the symptom is dose-dependent, increasing significantly with prolonged durations of computer use.[31] Subjects who spent more than 4 h using a computer display experienced more adverse CVS symptoms.[31] Our results indicated that VAS and CVS scores were significantly increased in individuals after smartphone use. Additionally, the smartphone group had higher fatigue, burning, and dryness scores at 4 h compared with the computer display group. Similar to the OSDI score, elevated VAS and CVS scores were associated with the decreased blink rate and frequent incomplete eye closure during smartphone use. Among subjective symptoms, the blurred vision and dullness did not change significantly after 4 h of smartphone use.

CVS is expected to be more severe after smartphone versus VDT uses. The smartphone screen is smaller, especially at horizontal scale, and viewed at a closer distance than other VDTs. Smartphones also have ergonomically lower positions than other VDTs. In general, the screen size of a smartphone is approximately 5 in, which is much smaller than that of other VDTs. Although blink intervals vary among individuals, the blink rate is approximately 20 blinks per minute in healthy individuals. VDT operation and other reading conditions caused significantly decrease in the blink rate; with less reduction in participants who read text on an expanded display.[26,32] To maintain stable and continuous vision, saccades are accompanied by visual suppression.[26] Thus, for small amplitude saccades, such as those involved in viewing a small screen, visual suppression is effective in stabilizing vision.[26] However, visual suppression in larger amplitude saccades is less effective and often requirescombined eye blink to maintain visual stability.[26] Thus, the eye blink rate under smartphone use is likely to be less than that under other VDTs`use due to visual suppression correlated with amplitude saccades. The preferred distance for viewing a mobile device (36.2 cm) is shorter than the typical distance for reading books (40 cm), with requirement fo greater accommodation and convergence.[33] Focusing on a smart mobile device screen may involve continuous accommodation efforts without blinking for an extended period.[34] Excessive smartphone use at a close reading distance and the resultant abnormalities in accommodation and vergence in adolescents can manifest as esotropia.[6] These differences in accommodation and convergence by distance may aggravate CVS in smartphone users.

Liquid-crystal display (LCD) and LED screens are useful and efficient in small portable electrical devices such as smartphones. Although health issues associated with VDT radiation are not guaranteed, electromagnetic radiation affects living tissue by destroying chemical bonds and charging neutral molecules.[35] LCD and LED screens also emit a large amount of blue light.[10] Many studies have reported harmful effects of blue light on the retina.[10] Blue light causes excessive ROS production and damages photoreceptor and retinal pigment epithelial cells.[10] In a previous study, we showed that overexposure to blue light decreased cellular viability and increased ROS production in human corneal epithelial cells.[9] Over exposure to blue light led to oxidative damage, apoptosis, and inflammation of the ocular surface resulting in dry eye, based on the findings of decreased FBUT and increased corneal fluorescein staining scores, terminal deoxynucleotidyl transferase nick end labeling-positive cells, inflammatory cytokines, and T-cells at the ocular surface.[9,10]

Among various oxidative stress markers, HEL is a good marker for oxidative modification as an early marker of lipid peroxidation.[36] In this study, our results indicated that the HEL concentration in tears was significantly increased at 4 h after smartphone use compared with that at baseline and 1 h after use, whereas it was not changed after computer display use; however, 4-HNE, MDA, and 8-OHdG concentrations showed no significantly change after use. Increased HEL levels may indicate smartphone use induced lipid peroxidation, a secondary reaction to ROS formation. 4-HNE and MDA are considered to be largely responsible for the cytopathological effects observed at the late phase of oxidative stress.[36,37] 8-OHdG is an oxidized derivative of deoxyguanosine and is a major DNA oxidation product.[38] With longer and more repetitive smartphone use, increased levels of other lipid peroxidation markers may also be observed. However, increasing the experimental time is challenging due to the probability of harmful effects. Several antioxidant protective mechanisms in the healthy ocular surface decrease function to ROS damage;[10] the observed differences in concentrations between the oxidation stress markers may reflect these mechanisms. In this study, DCF-DA assay was used to evaluate overall ROS production and cellular apoptosis.[10] Our results demonstrated an increased level of ROS production at the ocular surface after smartphone and computer display use, with higher value in the smartphone group at 4 h. These findings are consistent with previous reports on the relationship between visible light irradiation, cellular ROS levels and corneal epithelial cell viability *in vitro*.[9,39] Oxidative damage and apoptosis to the cornea, which may result from overexposure to blue light, may have association with inflammation of ocular surface and resultant dry eye.[10]

In summary, smartphone use aggravated subjective ocular symptoms and asthenopia; additional effects included compromised tear film stability and increased ROS production at the tear film and ocular surface. Smartphone-related changes at the ocular surface are multifactorial, and our study did not investigate all confounding factors. Smartphone use can deteriorate the tear film via the reduced rate of eye blink, incomplete closure of the eye, and exposure of the ocular surface. It can also induce the oxidative stress response at the ocular surface, thus aggravating ocular symptoms. In clinical practice, increased awareness of the tear film and ocular surface changes under smartphone use may enable clear understanding of the causes of ocular discomfort and management of ocular problems associated with excessive smartphone use.

## Supporting information

S1 Checklist TREND checklist. (DOCX)

S1 Protocol Trial protocol (English). (DOCX)

S2 Protocol Trial protocol (Korean). (DOCX)

S1 Supporting Information Questionnaire for assessment of subjective ocular symptoms and asthenopia (English). (DOCX)

S2 Supporting Information Questionnaire assessment of subjective ocular symptoms and asthenopia (Korean). (DOCX)
