# Supplementary material for: The influences of smartphone use on the status of the tear film and ocular surface
Source: PLoS One. 2018 Oct 31;13(10):e0206541. doi: 10.1371/journal.pone.0206541 (PMC6209417; doi:10.1371/journal.pone.0206541)
Supplement: S1 Protocol — (DOCX) [file pone.0206541.s002.docx]

**Clinical study protocol**

The Influences of Smartphone Use on the Status of the Tear Film and Ocular Surface

Protocol version: 1.2

Project Leader: Kyung Chul Yoon, M.D

1. Protocol title

The Influences of Smartphone Use on the Status of the Tear Film and Ocular Surface

2. Objective

To investigate the influences of smartphone use on ocular symptoms, status of the tear film, and oxidative stress in the tears and ocular surface

3. Background

The smartphone is one of the most innovative and remarkable inventions in recent decades and greatly impacts human life. Smartphones allow more varied activities including exploring web pages, watching video, chatting with a group, and enjoying social network services than the previous generation. Therefore, time spent gazing at display screens has increased with smartphone use compared with ordinary cellular phone use. One study reported that the average time spent using a smartphone nearly doubled from 98 minutes per day in 2011 to 195 minutes in 2013.

Because increased time of use is related to DED, excessive use of smartphones may affect the tear film and the ocular surface. Similar to office workers who spent more than 4 hours watching VDT experienced severe ocular symptoms, excessive smartphone use has been associated with multiple ocular symptoms. In addition, we recently demonstrated that blue light, which is emitted from the smartphone screen, harmfully affected human corneal epithelial cells. Overexposure to the blue light also deteriorated tear film and increased inflammatory markers and reactive oxygen species (ROS) production on the ocular surface of mice.

To our knowledge, no study has been published on ocular symptoms, signs or oxidative stress in the tears or the ocular surface associated with smartphone use. In the present study, we investigated the effects of smartphone use on subjective ocular asthenopia, tear film status, and oxidative marker levels in healthy subjects compared with computer display use.

4. Study design

Prospective, non-Randomized, Clinical Trial

5. Method

The healthy volunteers were assigned to either smartphone (n = 50) or computer display (n = 30; control) use. . In the smartphone group, subjects used the same smartphone with a 5.1 inch light emitting diode (LED) screen from the same manufacturer (Galaxy S6, Samsung, Seoul, South Korea). In the control group, subjects used the same computer display with a 19.0 inch screen (Samsung). The illumination intensity was fixed at 80% of the maximum brightness. The distance and angle between the screen of display and the subjects were also limited to be constant. All examinations were performed before smartphone use and after 1 hour and 4 hours of smartphone or computer display use by a single investigator. Each subject underwent a detailed ocular examinations including best corrected visual acuity, slit lamp biomicroscopy, OSDI score, VAS, Computer vision syndrome score, tear film BUT, Schirmer’s test, Keratograph 5M for NIKBUT and TMH, and history of additional usage of eyedrops which can affect ocular surface.

6. Inclusion criteria

(1) Healthy volunteer aged 20 to 40 years

(2) Healthy volunteer without any other ocular disease.

(3) Healthy volunteer use smartphone or computer display.

7. Exclusion criteria

(1) previous ocular surgery before the study

(2) active eye and periocular skin inflammation

(3) patients with pregnant woman

(4) previous ocular surgery before the study

(5) history of wearing contact lenses

(6) history of active treatment of eye drops (topical steroid or topical cyclosporin)

(7) systemic condition or medication that could cause any other ocular disease.

8. Statistics

Statistical Package for the Social Sciences software version 18.0 (SPSS, Inc, Chicago, IL) was used for all statistical analyses. Data are presented as the mean ± standard deviation. To assess changes in the various parameters before smartphone or computer display use and after 1 hour and 4 hours of use, Wilcoxon signed rank test was used. Mann-Whitney U test was used to compare the results between two groups. Differences were considered statistically significant if the p value was less than 0.05.

9. Outcome measurements

(1) Subjective symptom (OSDI, VAS, CVS score)

The OSDI questionnaire included the following subscales: (1) ocular symptoms (OSDI symptoms), (2) vision-related activities in daily living (OSDI visual function), and (3) environmental triggers (OSDI triggers). The total OSDI score and each subscale score, ranging from 0 to 100, were analyzed. Changes in ocular fatigue before and after smartphone use were examined by a VAS test to assess subjective asthenopia. We used a form with a scale of “I have no fatigue” (0) to “I feel extremely fatigued” (100) written on the extreme ends of a 100-mm line. We used a modified questionnaire proposed by Ames et al. to evaluate CVS score. These questionnaires included 5 questions on severity of fatigue, burning, dryness, blurred vision, and dullness associated with subjective asthenopia. Each question was graded on a numerical scale of 0–6, with 0 defined as none and 6 as most severe. All subjects completed the questionnaire before use and immediately after 1 hour and 4 hours of use.

(2) Tear film break-up time

Tear film BUT was evaluated 2 µl of 1% fluorescein solution was instilled on to the inferior palpebral conjunctiva after gentle depression of the lower eyelid. The interval between the last blink and the appearance of the first precorneal hypofluorescent spot, streak, or other irregularity interrupting the normal homogenous fluorescein pattern was recorded as the tear film BUT (seconds).

(3) Schirmer test

Schirmer’s test was performed by instilling one drop of proparacaine 0.5% anaesthetic, waiting for 5 min. A standard Schirmer test strip was then placed in the lateral canthus for another 5 min with the eyes closed. The length of wetting of the strip was measured using the millimeter scale.

(4) Keratograph 5M for NIKBUT and TMH

Non-invasive keratograph break-up (NIKBUT) and tear meniscus height (TMH) were measured by Keratograph® 5M (Oculus GmbH, Wetzlar, Germany). NIKBUT was evaluated by using an infrared diode video program in the Keratograph® 5M. The NIKBUT-average (the average time of all tear film breakups) was analyzed. TMH was evaluated by four infrared diodes with the red ring illumination deactivated and graded perpendicular to the lid margin at the 6 o’clock position of the midline of the cornea.

(5) Tear sampling

Basal tear samples were carefully obtained to avoid touching the ocular surfaces from the inferior tear meniscus of both eyes by using glass capillary tubes (Corning, Inc., Corning, NY) or micropipettes (Eppendorf, Hamburg, Germany) before smartphone use and immediately after 1 hour and 4 hours of use.

10. Referances

1. Yee RW, Sperling HG, Kattek A, et al. Isolation of the ocular surface to treat dysfunctional tear syndrome associated with computer use. Ocul Surf. 2007;5(4):308-15.

2. Nakaishi H, Yamada Y. Abnormal tear dynamics and symptoms of eyestrain in operators of visual display terminals. Occup Environ Med. 1999;56(1):6-9.

3. Kotegawa Y, Hara N, Ono K, et al. Influence of accommodative response and visual symptoms on visual display terminal adult operators with asthenopia through adequately corrected refractive errors. Nippon Ganka Gakkai Zasshi. 2008;112(4):376-81.

4. Kaido M, Kawashima M, Yokoi N, et al. Advanced dry eye screening for visual display terminal workers using functional visual acuity measurement: the Moriguchi study. Br J Ophthalmol. 2015;99(11):1488-92.

5. Aakre BM, Doughty MJ. Are there differences between ‘visual symptoms’ and specific ocular symptoms associated with video display terminal (VDT) use? Contact Lens Anterior Eye. 2007;30(3):174-82.

6. Schlote T, Kadner G, Freudenthaler N. Marked reduction and distinct patterns of eye blinking in patients with moderately dry eyes during video display terminal use. Graefes Arch Clin Exp Ophthalmol.2004;242(4):306-12.

7. Calvao-Santos G, Borges C, Nunes S, et al. Efficacy of 3 different artificial for the treatment of dry eye in frequent computer users and/or contact lens users. Eur J Ophthalmol. 2011;21(5):538-44.

8. Moon JH, Lee MY, Moon NJ. Association between video display terminal use and dry eye disease in school children. J Pediatr Ophthalmol Strabismus. 2014;51(2):87-92.

9. Kojima T, Ibrahim OM, Wakamatsu T, et al. The impact of contact lens wear and visual display terminal work on ocular surface and tear function in office workers. Am J Ophthalmol. 2011;152(6):933-940.

10. Wagner RS. Smartphones, video display terminals, and dry eye disease in children. J Pediatr Ophthalmol Strabismus. 2014;51(2):76.

11. Uchino Y, Uchino M, Yokoi N, et al. Alteration of tear mucin 5AC in office workers using visual display terminals: The Osaka Study. JAMA Ophthalmol. 2014;132(8):985-92.

12. Fenga C, Aragona P, Di Nola C, Spinella R. Comparison of ocular surface disease index and tear osmolarity as markers of ocular surface dysfunction in video terminal display workers. Am J Ophthalmol. 2014;158(1):41-48.

13. Ang CK, Mohidin N, Chung KM. Effects of wink glass on blink rate, nibut and ocular surface symptoms during visual display unit use. Curr Eye Res. 2014;39(9):879-84.

14. Walker PM, Lane KJ, Ousler GW 3rd. Diurnal variation of visual function and the signs and symptoms of dry eye. Cornea. 2010;29(6):607-12.

15. Miura DL, Hazarbassanov RM, Yamasato CK, et al. Effect of a light-emitting timer device on the blink rate of non-dry eye individuals and dry eye patients. Br J Ophthalmol. 2013;97(8):965-7.

16. Uchino M, Yokoi N, Uchino Y, et al. Prevalence of dry eye disease and its risk factors in visual display terminal users: the Osaka study. Am J Ophthalmol. 2013;156(4):759-66.

17. Uchino M, Schaumberg DA, Dogru M, et al. Prevalence of dry eye disease among Japanese visual display terminal users. Ophthalmology. 2008;115(11):1982-8.

18. Himebaugh NL, Begley CG, B Bradley A, Wilkinson JA. Blinking and tear breakup during four visual tasks. Optom Vis Sci. 2009;86(2):106-14.
