# Supplementary material for: The influences of smartphone use on the status of the tear film and ocular surface
Source: PLoS One. 2018 Oct 31;13(10):e0206541. doi: 10.1371/journal.pone.0206541 (PMC6209417; doi:10.1371/journal.pone.0206541)
Supplement: S2 Protocol — (DOCX) [file pone.0206541.s003.docx]

**연구계획서**

**1. 연구의 명칭 및 단계**

스마트폰 사용이 눈물층과 안구표면에 미치는 영향

**2. 연구의 실시기관명 및 주소**

전남대학교병원, 광주광역시 동구 제봉로 42

**3. 연구의 책임자, 담당자, 공동연구자 및 관리약사의 성명 및 직명**

책임자: 윤경철(교수)

공동연구자 및 연구원 : 최원(전임의사), 최지숙(연구간호사), 김영휘(레지던트)

**4. 연구비 지원기관명 및 주소(모니터 직명 및 성명** 포함)

해당사항 없음.

**5. 연구의 목적 및 배경**

기존의 여러 연구에서 영상 표시 장치 (Video display terminal) 의 사용이 건성안 (Dry eye disease) 에 미치는 영향이 보고되었다. 건성안은 가장 흔한 안과 질환 중 하나로, 눈물막의 불안정성 (tear film instability) 이 발생하여 안구 표면에 손상을 일으킬 수 있는 눈물과 안구 표면의 다인성 질환이며, 눈물막의 오스몰농도의 증가와 안구표면의 염증을 동반한다고 알려져 있다.

영상 표시 장치가 일상생활에 널리 확대되어 사용되면서 이와 관련된 사용자들의 안구 불편감 및 건성안 증상도 함께 많아지게 되었으며, 여러 연구에서 눈물층의 역동학과 노출된 안구표면의 상관관계 등을 보고하였다. 또한 영상 표시 장치의 사용이 지속적인 눈의 집중을 요구하기 때문에 눈깜박임 (blinking) 횟수가 감소하게 되어 안구 표면의 건조를 증가시켜 증상이 악화된다고 알려져 있다.

스마트폰은 영상 표시 장치의 한 종류로, 특정 직업과 관련없이 모든 직종의 인구집단에서 널리 보급되어 있으며 스마트폰과 눈물막과 안구표면의 역동학에 관한 연구는 아직까지 많지 않다.

따라서 본 연구에서는 스마트폰의 사용 시간이 눈물막과 안구표면에 어떤 영향을 미치는지 알아보고자 한다.

**6. 예상연구기간**

IRB 승인일로부터~ 12개월 간

**7. 연구방법**

① 연구방법개요

(1) 50 명의 일반인을 대상으로 스마트폰 사용 전 및 사용 후 1 시간, 4 시간째의 ocular surface parameter 를 검사한다. 스마트폰은 아이폰 (Apple Inc., Cupertino, CA, USA) 을 사용하며 스마트폰을 이용한 작업은 가장 밝은 밝기의 80% 하에 이용하는 것으로 한다. 각 대상자들은 실내 조도 500~1500 Lux, 섭씨온도 25℃, 습도 40~50% 의 동일한 조건의 환경에서 스마트폰 작업을 수행한다. 각 군 모두 스마트폰을 사용하기 전 (base line) 과 사용 후 Visual analogue scale, OSDI score, VAS, Computer vision syndrome & asthenopia related ocular symptoms, Ocular fatigue index, Tear film & ocular surface parameters : Tear film breakup time (BUT), Schirmer test, Corneal staining (KEP), Non-invasive BUT (NIBUT, Keratograph), Tear meniscus height (TMH, Keratograph), 및 Tear-film analysis (Anti-oxidant enzyme, Lipid peroxidation marker) 를 측정한다.

② 연구대상자의 선정기준, 제외기준

연구대상자 선정은 외래에 내원하는 대상자에게 연구에 대해 설명 후 지원자를 모집한다.

(연구 설명문을 제시하여 연구의 목적 및 방법, 연구 도중 발생가능한 문제 등에 대하여 충분히 설명 후 동의를 얻은 대상자에게 한한다.) 대상자 모집 시 대상자에게 다음과 같은 항목들을 체크하게 한 후 제외기준에 해당하는 항목이 하나라도 있을 경우 대상자에서 제외한다.

(1) 선정기준

1. 특별한 안과적 질환의 기왕력이 없는 스마트폰 사용자

2. 연령은 20-40세를 대상으로 한다.

3. 이전 안과적 수술 기왕력이 없는 자

(2) 제외기준

1. 이전 안과적 수술 기왕력이 있는 자

2. 기존에 안구 건조증으로 약물치료를 시행 받고 있는 자

(인공눈물만을 사용하는 경우도 포함)

3. 확장성 각막변성 및 각막이영양증 등을 비롯한 안구표면에 영향을 미칠 수 있는 다른 안과적 질환이 있는 경우

4. 콘택트렌즈 사용자

③ 목표 연구대상자의 수 및 산출 근거

2016년 3월부터 2016년 4월까지 본원에 내원한 80명을 대상으로 한다. 산출근거는 기존문헌을 참고하여 의의 있는 연구 결과를 낼 수 있는 대상자 수가 40명 이상이기 때문이다. 따라서 기존 유사연구를 참조하여 총 80명을 대상으로 한다.

(참고문헌)

Yee RW, Sperling HG, Kattek A, et al. Isolation of the ocular surface to treat dysfunctional tear syndrome associated with computer use. Ocul surf. 2007;5(4):308-15.

④ 관찰항목, 관찰검사방법 및 임상검사항목

1. Visual analogue scale (Ocular fatigue / Dry eye symptom


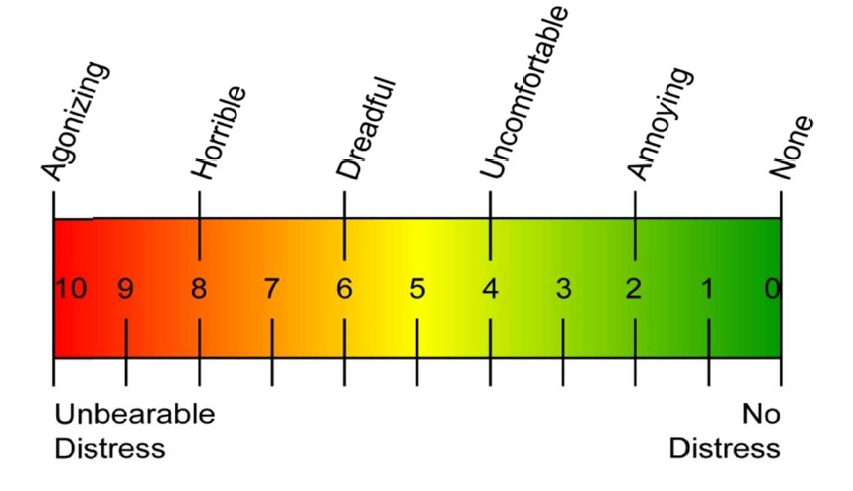


2. OSDI score (0-100)

* Subscales

(0-None, 4-all of the time)

a. Ocular symptoms

1. Eyes that are sensitive to light?

2. Eyes that feel gritty?

3. Painful or sore eyes?

4. Blurred vision?

5. Poor vision?

b. Vision-related activity

1. Reading?

2. Driving at night?

3. Working with a computer or bank machine (ATM)?

4. Watching TV?

c. Environmental trigger

1. Windy conditions?

2. Places or areas with low humidity (very dry)?

3. Areas that are air conditioned?

3. Computer vision syndrome & Aeshenopia related ocular symptoms

(0- No symptoms, 6-very severe)

(1) Fatigue (2) Burning (3) Dryness (4) Blurred vision (5) Dullness

4. Tear film & ocular surface parameters

1) Tear film breakup time

2) Schirmer test (with anesthesia – BST)

3) Corneal staining, keratoepitheliopathy (KEP)

4) Keratograph – NIKBUT

5) Keratograph – TMH

6. Tear film analysis

Tear collection 을 통해 tear 의 성분 중 anti-oxidant enzyme (SOD1), Lipid

peroxidation marker 인 HEL, 4HNE, MDA, 8ODHG 의 농도를 분석한다.

⑤ 통계분석 원칙 및 방법

SPSS 18.0을 이용하여 각 군의 정규성 검정을 위해 Kolmogorov-Smirnov test를 시행한다.

80명의 스마트폰 및 컴퓨터 디스플레이 사용 전 baseline과 사용 후 1시간 및 4시간 후의 실험결과값의 차이를 비교하기 위해 paired t test (또는 Wilcoxon signed-rank test) 및 두 군간의 차이를 비교하기위해 Student t-test (또는 Mann Whitney U-test) 를 사용한다. P 값이 0.05 미만인 경우를 통계적으로 유의하다고 정의한다.

9. 연구의 윤리성 확보를 위한 방안

본 실험은 1964 년 핀란드 헬싱키에서 개최된 세계의사협회 제 18 차 총회에서 제정 채택된 의사윤리와 임상시험에 관한 기본 준칙을 준수한다. 환자의 정보가 노출될 수 있는 데이터는 기밀문서함에 보관하며 전산화된 데이터는 보안을 기해 해당자를 제외하고는 접근을 금지시킨다.

10. 참고 문헌

1. Yee RW, Sperling HG, Kattek A, et al. Isolation of the ocular surface to treat dysfunctional tear syndrome associated with computer use. Ocul Surf. 2007;5(4):308-15.

2. Nakaishi H, Yamada Y. Abnormal tear dynamics and symptoms of eyestrain in operators of visual display terminals. Occup Environ Med. 1999;56(1):6-9.

3. Kotegawa Y, Hara N, Ono K, et al. Influence of accommodative response and visual symptoms on visual display terminal adult operators with asthenopia through adequately corrected refractive errors. Nippon Ganka Gakkai Zasshi. 2008;112(4):376-81.

4. Kaido M, Kawashima M, Yokoi N, et al. Advanced dry eye screening for visual display terminal workers using functional visual acuity measurement: the Moriguchi study. Br J Ophthalmol. 2015;99(11):1488-92.

5. Aakre BM, Doughty MJ. Are there differences between ‘visual symptoms’ and specific ocular symptoms associated with video display terminal (VDT) use? Contact Lens Anterior Eye. 2007;30(3):174-82.

6. Schlote T, Kadner G, Freudenthaler N. Marked reduction and distinct patterns of eye blinking in patients with moderately dry eyes during video display terminal use. Graefes Arch Clin Exp Ophthalmol.2004;242(4):306-12.

7. Calvao-Santos G, Borges C, Nunes S, et al. Efficacy of 3 different artificial for the treatment of dry eye in frequent computer users and/or contact lens users. Eur J Ophthalmol. 2011;21(5):538-44.

8. Moon JH, Lee MY, Moon NJ. Association between video display terminal use and dry eye disease in school children. J Pediatr Ophthalmol Strabismus. 2014;51(2):87-92.

9. Kojima T, Ibrahim OM, Wakamatsu T, et al. The impact of contact lens wear and visual display terminal work on ocular surface and tear function in office workers. Am J Ophthalmol. 2011;152(6):933-940.

10. Wagner RS. Smartphones, video display terminals, and dry eye disease in children. J Pediatr Ophthalmol Strabismus. 2014;51(2):76.

11. Uchino Y, Uchino M, Yokoi N, et al. Alteration of tear mucin 5AC in office workers using visual display terminals: The Osaka Study. JAMA Ophthalmol. 2014;132(8):985-92.

12. Fenga C, Aragona P, Di Nola C, Spinella R. Comparison of ocular surface disease index and tear osmolarity as markers of ocular surface dysfunction in video terminal display workers. Am J Ophthalmol. 2014;158(1):41-48.

13. Ang CK, Mohidin N, Chung KM. Effects of wink glass on blink rate, nibut and ocular surface symptoms during visual display unit use. Curr Eye Res. 2014;39(9):879-84.

14. Walker PM, Lane KJ, Ousler GW 3rd. Diurnal variation of visual function and the signs and symptoms of dry eye. Cornea. 2010;29(6):607-12.

15. Miura DL, Hazarbassanov RM, Yamasato CK, et al. Effect of a light-emitting timer device on the blink rate of non-dry eye individuals and dry eye patients. Br J Ophthalmol. 2013;97(8):965-7.

16. Uchino M, Yokoi N, Uchino Y, et al. Prevalence of dry eye disease and its risk factors in visual display terminal users: the Osaka study. Am J Ophthalmol. 2013;156(4):759-66.

17. Uchino M, Schaumberg DA, Dogru M, et al. Prevalence of dry eye disease among Japanese visual display terminal users. Ophthalmology. 2008;115(11):1982-8.

18. Himebaugh NL, Begley CG, B Bradley A, Wilkinson JA. Blinking and tear breakup during four visual tasks. Optom Vis Sci. 2009;86(2):106-14.

11. 연구대상자 설명문 및 동의서 :

**연구대상자 설명문**

1. 임상 연구 제목

스마트폰 사용이 눈물층과 안구표면에 미치는 영향

2. 연구 책임자

전남대학교병원 안과 윤경철

3. 개요

본 연구는 스마트폰 및 컴퓨터디스플레이 사용 시간에 따른 안과 검사, 즉 눈꺼풀 피부, 눈물막과 각결막을 포함한 안구표면의 구체적인 변화에 대한 연구를 통해 그 영향을 확인하고자 하는 연구입니다. 이 연구를 수행하는 전남대학교병원 소속 윤경철 (062-220-6741) 교수 또는 최원 (062-220-6758) 전임의가 귀하에게 이 연구 참여 과정에 대하여 설명해 줄 것입니다. 이 연구는 자발적으로 참여 의사를 밝히신 분에 한하여 수행 될 것이며, 귀하께서는 본 임상연구에 참여 의사를 결정하기에 앞서, 임상연구가 왜 수행되고, 귀하의 정보가 어떻게 사용될지, 본 임상연구가 어떤 것을 포함하고 있는 지와 가능한 이점, 위험, 불편함은 무엇인지에 대하여 이해하는 것이 중요합니다. 다음의 설명을 신중하게 시간을 가지고 주의 깊게 읽으시기 바라며, 필요하시면 귀하의 주치의 또는 가족이나 친구들과 상의하시기 바랍니다. 만일 어떠한 질문 사항이 있으시면 담당 연구원이 자세하게 설명해 줄 것입니다.

4. 임상연구의 목적

이 연구의 목적은 스마트폰 사용시간에 따른 안과적 검사, 즉 눈꺼풀 피부, 눈물막과 각결막을 포함한 안구표면의 구체적인 변화에 대한 연구를 통해 그 영향 및 효과를 확인하고자 하는 것입니다.

5. 연구기기

Galaxy S6 (Samsung, Seoul, South Korea) 및 LCD 모니터(computer display with a 19.0 inch screen, Samsung) 이 연구에 포함됩니다.

6. 임상연구 방법에 관한 설명

본 임상연구는 연구 대상자의 배경 및 병력조사, 간단한 신체검사 등의 검사를 통해 연구대상자 선정기준 및 제외기준에 적합한 대상에 한해 실시합니다. 연구 대상자는 80명이며, 연구참여에 동의하시면, 선정/제외기준을 확인하고, 연구대상자로 선정되면 다음과 같이 진행됩니다. 스마트폰 사용 전에 약 20분 동안 다음과 같은 검사를 시행합니다.

1. 시각유사척도 (Visual analogue scale)
2. 눈물막파괴시간 (Tear break up time)

연구대상자의 눈에 플루레신 염색약을 묻히고 세극등의 코발트블루 필터를 사용하여 눈을 감게한 후 뜬 상태로 정면을 주시하게 하여 눈물층이 깨지는 데 걸리는 시간을 측정합니다.

1. 기본눈물분비량 검사 (Schirmer` test)

Alcaine 점안마취제 사용 후 Schirmer 검사용지를 아래 눈꺼풀 가쪽 1/3 지점에 눈

꺼풀결막에 걸쳐놓고 3분 후 용지를 떼어 눈물의 분비량을 mm 단위로 읽습니다.

1. 각막 및 결막염색점수 (corneal and conjunctival staining score)

각막 및 결막염색점수는 fluorescein dye 및 lissamine green dye를 통하여 각막 및 결막염색점수를 평가합니다.

1. 눈물층 성분 분석 (Tear film analysis )

대상자의 눈물을 채취하여 눈물층의 성분을 분석합니다.

이후 지급받은 스마트폰을 1시간 사용 후 그리고 4시간 사용 후, 위와 같은 검사를 각각 시행합니다.

7. 임상연구 제한 사항 및 연구대상자 의무

연구기간 내에는 부득이하게 연구참여를 할 수 없을 경우 반드시 연구자에게 보고하여야 합니다.

8. 연구대상자에게 예견되는 부작용, 위험과 불편함

- 스마트폰 및 컴퓨터 디스플레이 의 장기간 사용에 따른 눈깜박임감소에 따른 안구건조감, 근거리작업의 장기화에 따른 과도한 눈조절 등에 의한 피로감 등이 발생할 수 있습니다. 그러나 본 연구에 참여함으로 인해 일시적으로 발생할 수 있는 안구 불편감 등에 의해 일상생활의 지장은 발생하지 않을 것으로 생각됩니다. 또한 설문 및 각종 검사를 진행함에 따른 불편감이 발생할 수 있으므로 이 점에 대해서는 양해바랍니다.

9. 연구대참여로 인한 비용

연구용 검사에 필요한 비용은 전적으로 연구자가 부담하므로 연구에 참여하게 되는 대상자 는 연구용 검사에 필요한 비용은 따로 부담하지 않아도 됩니다.

10. 연구대상자에게 예견되는 이득

본 임상 시험에 참여 함으로서 귀하에게 직접적 혜택이 보장되는 것은 아닙니다.

11. 금전적 지급

본 임상연구의 연구대상자로 선정되어 연구에 참여하는 경우 연구 종료 후 교통비 10만원 이 지급됩니다. 본 임상연구에 참여하기 위해 연구대상자가 지불할 비용은 없습니다. 그러나 본 임상연구과 무관한 입원비 및 검사비, 진찰비는 본인이 부담하도록 합니다.

12. 연구 관련 새로운 정보의 지속적 제공

본 연구 기간 중 귀하의 연구에 참여 여부를 결정하는데 영향을 줄 수 있는 새로운 유의한 정보가 얻게 되는 즉시 귀하 또는 귀하의 대리인에게 알려 드릴 것입니다.

13. 피해발생 시 연구대상자 보상 (의료적 치료/보상)

본 임상연구 기간 중 연구담당자는 피험자의 안전에 만전을 기할 것이며 예측 가능한 부작용 이외의 심각한 부작용 발생시 신속하고 적절한 조치를 취하여 가능한 그 부작용을 최소화할 것입니다.

14. 비밀 보장

연구대상자의 신원을 파악할 수 있는 기록은 비밀로 보장될 것이며, 임상연구의 결과가 출판될 경우 연구대상자의 신원은 익명상태로 유지됩니다.

15. 자발적 참여

본 임상연구에 참여하시는 것은 귀하에게 달려 있습니다. 귀하는 언제든지 연구에 참여하지 않기로 결정할 수 있고 또한 연구를 그만 둘 수 있습니다. 귀하가 본 연구에 참여하지 않아도 아무런 불이익을 받지 않으며 귀하의 결정은 향후 귀하가 진료를 받는 것에 영향을 미치지 않습니다.

16. 임상연구 관련 책임자 및 연락처

귀하는 연구책임자 (윤경철/062-220-6741) 및 연구담당자 (최원/062-22-6758) 에게 임상연구 기간 중에 언제든지 추가적인 정보를 요청할 수 있습니다. 또한 귀하는 연구대상자로서의 귀하의 권리에 대해 의문이 있을 경우 전남대학교병원 생명의학연구윤리심의위원회(전화번호 062-220-5257)로 문의하실 수 있습니다.

**연구대상자 동의서**

1. 본인은 임상연구에 대해 구두로 설명을 받고 상기 연구대상자 설명문을 읽었으며 담당 연구원과 이에 대하여 의논하였습니다.

2. 본인은 위험과 이득에 관하여 들었으며 나의 질문에 만족할 만한 답변을 얻었습니다.

3. 본인은 이 연구에 참여하는 것에 대하여 자발적으로 동의합니다.

4. 본인은 이후의 치료에 영향을 받지 않고 언제든지 연구의 참여를 거부하거나 연구의 참여를 중도에 철회할 수 있고 이러한 결정이 나에게 어떠한 해가 되지 않을 것이라는 것을 알고 있습니다.

5. 본인은 이 설명서 및 동의서에 서명함으로써 의학 임상연구 목적으로 나의 개인정보가 현행 법률과 규정이 허용하는 범위 내에서 연구자가 수집하고 처리하는데 동의합니다.

6. 본인은 연구대상자 설명문과 동의서 사본을 받을 것을 알고 있습니다.

연구대상자 성명 서명 날짜 (년/월/일)

동의서 받은 연구원 성명 서명 날짜 (년/월/일)

연구책임자 성명 서명 날짜 (년/월/일)
