# Supplementary material for: The influences of smartphone use on the status of the tear film and ocular surface
Source: PLoS One. 2018 Oct 31;13(10):e0206541. doi: 10.1371/journal.pone.0206541 (PMC6209417; doi:10.1371/journal.pone.0206541)
Supplement: S1 Supporting Information — (DOCX) [file pone.0206541.s004.docx]

Questionnaire

I. Visual analogue scale

I feel extremely fatigued

I have no fatigue

|  |  |  |  |  |  |  |  |  |  |
| --- | --- | --- | --- | --- | --- | --- | --- | --- | --- |
|  |  |  |  |  |  |  |  |  |  |

**100**

**90**

**80**

**70**

**60**

**50**

**40**

**30**

**20**

**10**

**0**

|  | Baseline | After 1 hour | After 4 hours |
| --- | --- | --- | --- |
| VAS |  |  |  |

2. OSDI score

|  | Baseline | After 1 hour | After 4 hours |
| --- | --- | --- | --- |
| OSDI |  |  |  |

* Subscales (0-None, 4-all of the time)

A. Ocular symptoms

1. Eyes that are sensitive to light?

2. Eyes that feel gritty?

3. Painful or sore eyes?

4. Blurred vision?

5. Poor vision?

B. Vision-related activity

1. Reading?

2. Driving at night?

3. Working with a computer or bank machine (ATM)?

4. Watching TV?

C. Environmental trigger

1. Windy conditions?

2. Places or areas with low humidity (very dry)?

3. Areas that are air conditioned?

3. Computer vision syndrome, aesthenopia related ocular symptoms

(0- No symptoms, 6-very severe)

|  | Baseline | After 1 hour | After 4 hours |
| --- | --- | --- | --- |
| Fatigue |  |  |  |
| Burning |  |  |  |
| Dryness |  |  |  |
| Blurred vision |  |  |  |
| Dullness |  |  |  |
