# Supplementary material for: The influences of smartphone use on the status of the tear film and ocular surface
Source: PLoS One. 2018 Oct 31;13(10):e0206541. doi: 10.1371/journal.pone.0206541 (PMC6209417; doi:10.1371/journal.pone.0206541)
Supplement: S2 Supporting Information — (DOCX) [file pone.0206541.s005.docx]

설 문 지

I. Visual analogue scale

“극도로 피로하다”

“전혀 피로하지 않음”

|  |  |  |  |  |  |  |  |  |  |
| --- | --- | --- | --- | --- | --- | --- | --- | --- | --- |
|  |  |  |  |  |  |  |  |  |  |

**100**

**90**

**80**

**70**

**60**

**50**

**40**

**30**

**20**

**10**

**0**

|  | 사용 전 | 사용 후 1시간 | 사용 후 1시간 |
| --- | --- | --- | --- |
| VAS |  |  |  |

2. OSDI score

|  | 사용 전 | 사용 후 1시간 | 사용 후 1시간 |
| --- | --- | --- | --- |
| OSDI |  |  |  |

* Subscales (0-None, 4-all of the time)

A. 안구 인자

1. 눈이 빛에 예민하다

2. 눈에 모래가 들어간 느낌이다.

3. 눈이 따갑다

4. 시야가 흐리다.

5. 앞을 보기가 힘들다.

B. 시각 관련 인자

1. 책 읽기

2. 야간 운전

3. 컴퓨터 or 현금인출기 사용

4. TV 시청

C. 환경적 인자

1. 바람 부는 날씨

2. 건조한 장소

3. 에어컨이 켜진 장소

3. Computer vision syndrome, aesthenopia related ocular symptoms

(0- No symptoms, 6-very severe)

|  | 사용 전 | 사용 후 1시간 | 사용 후 1시간 |
| --- | --- | --- | --- |
| 피로감 |  |  |  |
| 작열감 |  |  |  |
| 건조감 |  |  |  |
| 흐릿한 시야 |  |  |  |
| 둔한 느낌 |  |  |  |
